# Identification of Potential Phytochemical Inhibitors From Conium maculatum Targeting the Epidermal Growth Factor Receptor in Metastatic Colorectal Cancer via Molecular Docking Analysis

**DOI:** 10.7759/cureus.48000

**Published:** 2023-10-30

**Authors:** Samyuktha Venkateswaran, Hema Priya Manivannan, Arul Prakash Francis, Vishnu Priya Veeraraghavan, Gayathri R, Kavitha Sankaran

**Affiliations:** 1 Centre of Molecular Medicine and Diagnostics (COMManD) Department of Biochemistry, Saveetha Dental College and Hospitals, Saveetha Institute of Medical and Technical Sciences, Saveetha University, Chennai, IND

**Keywords:** mortality, novel compound, biomedicine, cancer, phytochemicals

## Abstract

Background

Metastatic colorectal cancer (mCRC) continues to rank as the second deadliest cancer on the global scale. CRC diagnosed at metastatic (stage IV) makes treatment strategies more challenging. Even though there are numerous therapeutic options available, the side effects of these treatments threaten the human health. Therefore, we are in the phase of searching new molecules that are less harmful and cost-effective. The common source of many pharmaceutical medications is plants. This study focuses on virtually screening phytochemicals from *Conium maculatum *as potential inhibitors of the epidermal growth factor receptor (EGFR), a crucial target in cancer therapy.

Methods and materials

*C. maculatum* was selected due to its phytochemicals and prior indications of its anticancer properties. In silico investigations encompass druglikeness screening, pharmacokinetics assessment, molecular docking, toxicity prediction, molecular target screening, and molecular dynamics simulations. A comprehensive analysis led to the identification of promising lead compounds.

Results

A total of 25 compounds exhibited favorable pharmacokinetic and drug-like characteristics. Among them, 12 compounds displayed a high affinity for EGFR as determined by molecular docking experiments. Further safety assessment using ProTox-II revealed that seven compounds had no anticipated toxicity, affirming their safety profiles.

Conclusion

These findings collectively predicted the efficacy of seven phytochemicals from *C. maculatum* as EGFR inhibitors in mCRC. Further experimental investigations and optimization of the identified leads were needed to validate the efficacy and safety of identified lead compounds and explore their therapeutic potential in CRC.

## Introduction

Colorectal cancer (CRC), a prevalent cancer that originates in the colon or rectum, is one of the foremost causes of cancer-related deaths around the world. In terms of therapies and survival of patients, metastatic CRC (mCRC), which is referred to as the spreading of cancer beyond the primary tumor site, offers substantial difficulties [1]. Millions of people globally have been diagnosed with CRC. In 2020, 1.9 million new CRC cases were recorded, with 0.9 million deaths. CRC is the second most fatal malignancy worldwide. According to estimations, there might be up to 3.2 million CRC cases by 2040 [2,3].

Almost 20% of people diagnosed with CRC are metastatic (stage IV), making treatments for this condition more challenging and intricate [4]. The percentage of mCRC patients with survival times of more than a year is 70% to 75%. Even though there are numerous therapeutic options for mCRC, including combination chemotherapy, surgery, and antibody therapy, the side effects of these treatments threaten the human health [5]. Moreover, these treatments are exceptionally costly. Therefore, it is essential to look for molecules that are less harmful and cost-effective. The common source of numerous pharmaceutical medications is plants, and many chemotherapy medications were derived from plant sources. Plants have medicinal qualities that could be used to treat various illness problems [6]. The pharmacological ingredients in the complementary and alternative medicine (CAM) systems, including homeopathy, Ayurveda, and Siddha, are plant based. Numerous illness conditions can be treated with drugs made from bioactive substances found in plants [7,8].

Nearly 25-75% of individuals with colorectal disease have elevated levels of the membrane-bound tyrosine kinase growth factor receptor protein known as epidermal growth factor receptor (EGFR). CRC metastases and poor prognosis are linked to the overexpression of EGFR [9,10]. Targeting EGFR and its inhibition can prevent cancer growth by inducing apoptosis. Buzard et al. [11] investigated the role of osimertinib, an EGFR targeted drug in a patient with metastatic sigmoid colon adenocarcinoma. The treatment for seven months resulted in an improvement in hepatic metastases [11]. Bioinformatics plays a critical role in drug design, facilitating the discovery and development of new therapeutic agents in a more efficient and targeted manner. Bioinformatics approaches have assumed a central role in expediting drug discovery. Among these approaches, molecular docking and drug-likeness screening have become indispensable components of computer-aided drug design (CADD), offering unprecedented insights into the rational design of novel drug candidates. These advanced machine-learning techniques have revolutionized various aspects of drug discovery and development [12,13]. 

The therapeutic plant* Conium maculatum*, often referred to as poison hemlock, has a long tradition in conventional medicine. It comprises many kinds of phytochemicals that include alkaloids, flavonoids, and polyacetylenes that could provide medicinal properties. *C. maculatum* extracts have been demonstrated in many studies to exhibit anticancer activity in multiple kinds of cancer and have the potential as a source of novel therapeutic agents. Its ethanolic extract has previously been reported for anticancer activity against cancer cell lines [14].

*C. maculatum* is a plant used in homeopathic remedies that is utilized for the treatment of various medical conditions, including cancer. In a study conducted by Bishayee et al. [14] in 2012, it was observed that an extract derived from *C. maculatum*, specifically the mother tincture used in homeopathy, exhibited notable anticancer properties when tested against HeLa cell lines. The mechanism underlying this anticancer activity was attributed to the initiation of reactive oxygen species (ROS)-mediated DNA damage, ultimately leading to cell death through apoptosis. Remarkably, the same *Conium* extract demonstrated a lower level of cytotoxicity when applied to normal cells [14]. Given the rich traditional history of the usage of this plant in homeopathy, we have chosen *C. maculatum *as the subject of our study. *C. maculatum* has been documented in John Henry Clarke's Materia Medica as a remedy with historical usage for cancer treatment [15]. Building upon the promising results obtained from previous research, we have specifically selected this plant for our investigation. Indeed, while *C. maculatum* has a historical background in homeopathic applications, its potential utility in mainstream cancer management requires a robust and evidence-based assessment. Our study, in this context, aims to contribute to this endeavor by providing valuable insights into the pharmacological properties and potential anticancer effects of *C. maculatum*. By combining traditional knowledge with modern scientific investigation, we aim to provide a more comprehensive understanding of its eligibility for use in the treatment of cancer.

The primary objective of our study is to conduct a preliminary exploration into the phytochemicals of *C. maculatum* and assess the drug-like properties and potential pharmacokinetic attributes and its possible EGFR inhibitory activity in mCRC. Thus, we have analyzed these phytochemicals' interactions and binding affinities with the EGFR active site using molecular docking. Identifying effective EGFR inhibitors in *C. maculatum* might provide important insights into creating innovative therapeutic drugs for treating mCRC.

## Materials and methods

Proteins and ligands

The phytochemicals present in *C. maculatum* were obtained from Dr. Duke’s Phytochemical and Ethnobotanical Database (https://phytochem.nal.usda.gov/) [16]. This database provides comprehensive information on the phytochemical composition of various plants, including *C. maculatum*. The 2D, 3D, and canonical simplified molecular-input line-entry system (SMILES) representations of the selected phytochemicals from *C. maculatum *were retrieved from the PubChem database (https://pubchem.ncbi.nlm.nih.gov/). PubChem is a vast public repository that contains chemical information, including structures and properties, for a wide range of compounds [17]. Phytochemicals that were not available in the PubChem database were excluded from the study. This step ensures that only compounds with existing structural information and associated properties are considered for further analysis. A total of 27 phytochemicals from *C. maculatum* were selected and used for this study. The selection was based on their availability in the PubChem database and their relevance to potential anticancer properties reported in the literature. Regorafenib, a known drug used in the treatment of mCRC, was included as a control in the study. The three-dimensional structure of the EGFR protein was retrieved from Research Collaboratory for Structural Bioinformatics Protein Data Bank (RCSB PDB)(https://www.rcsb.org/). The PDB ID for EGFR was 1XKK.

Druglikeness and pharmacokinetics

The druglikeness and pharmacokinetic properties of the 27 compounds, as well as a control drug, were evaluated using the SwissADME online web server (http://www.swissadme.ch/) [18]. The evaluation of the druglikeness was performed based on Lipinski's rule of five, which includes criteria assuring that the compounds have a molecular weight of 500 Daltons or less, five or fewer hydrogen bond donors, 10 or fewer hydrogen bond acceptors, and a computed logarithm of the partition coefficient (log P) of 5 or less. We aimed to identify compounds with favorable oral bioavailability and drug-like characteristics. The pharmacokinetic characteristics of compounds, including absorption in the gastrointestinal tract (GIA), blood-brain barrier (BBB) permeability, propensity to act as a substrate for p-glycoprotein, and interactions with certain cytochrome P450 isoenzymes, were examined. These evaluations gave important insights into the pharmacokinetic characteristics of the substances, which showed their probable distribution and metabolism inside the body. The SMILES of the compounds- simplified molecular notations that contain the structural characteristics of the compounds were obtained from PubChem to predict druglikeness and pharmacokinetics. These SMILES were then submitted to the SwissADME web server for analysis [19].

Molecular docking

Molecular docking is a crucial method that aids in determining a ligand's affinity to a particular target, which makes it easier to develop novel medications. The target protein in this investigation was the EGFR. Using AutoDockTools version 1.5.7 (The Scripps Research Institute, USA), as described by Roy et al. [20] in their article, the docking procedure was carried out. Before the docking process, the target EGFR was prepared by eliminating water molecules, adding hydrogen bonds, and computing Gasteiger charges. The ligands were treated as flexible entities to allow for conformational changes during the docking procedure. Grid coordinates for docking were established using the inhibitor already present in the EGFR receptor. The ligands were docked with the EGFR to produce a variety of conformations. The conformation with the lowest binding affinity was chosen as the most likely binding position among them. BIOVIA Discovery Studio Visualizer version 21.1.0.20298 (Dassault Systèmes, France) was used to visualize viewing and examining the docked positions.

Toxicity analysis

Utilizing the ProTox II online web server (https://tox-new.charite.de/protox_II/) [21], the compounds with binding affinities ranging from -6.5 to -8 kcal/mol were analyzed for their toxicological characteristics. This was done to identify any potential risks associated with compounds. The evaluation included hepatotoxicity, carcinogenicity, immunogenicity, mutagenicity, and cytotoxicity as outcomes. Compounds with no predicted toxicity were considered as leads, and the identified leads were analyzed further.

Molecular dynamics simulations

The flexibility of the ligand-target complex was assessed using the CABS-flex 2.0 web server. The result of the protein flexibility analysis was represented by the root mean square fluctuation (RMSF). Protein flexibility simulations can be done quickly and effectively with the CABS-flex server. High-resolution protein dynamics simulations using CABS-flex are possible, capturing nearly native protein dynamics over a 10-ns timeframe [22]. Because of these capabilities, it is especially helpful for rapid assessments of the stability of protein-ligand interactions. For the 50 simulation cycles of the CABS-flex simulations, default parameters were used.

Bioavailability radar and molecular target prediction

The identification of lead compounds based on their toxicological profiles was further analyzed for their bioavailability using the SwissADME web server (http://www.swisstargetprediction.ch/) [23]. In order to analyze the lead compound's potential molecular targets, the SwissTargetPrediction web server was utilized; these predictions aid in understanding the compound’s pharmacological activities and their potential therapeutic applications [24].

## Results

Proteins and ligands

**Figure 1:**
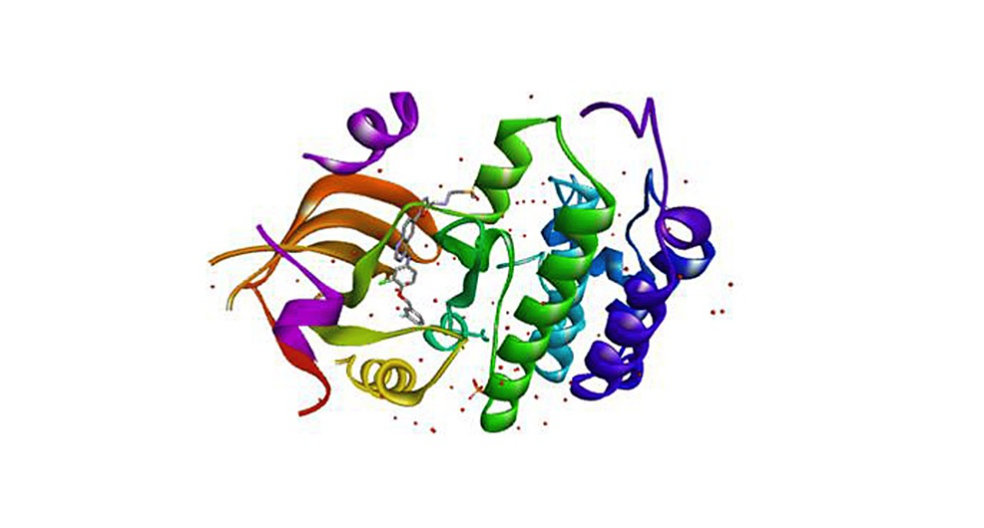
3D structure of target protein (Protein Data Bank (PDB) ID: 1XKK)

The selected list of phytochemicals from *C. maculatum* is given in Table 1. Figure 1 illustrates the target EGFR's 3D structure obtained from the PDB.

**Table 1:** List of selected phytochemicals from Conium maculatum

No	Compound
1	Arabinose
2	Caffeic acid
3	Chlorogenic acid
4	Conhydrine
5	Coniine
6	Diosmin
7	Falcarinol-one
8	Falcarinone
9	Gamma- Coniceine
10	Pseudoconhydrine
11	Myrcene
12	Bicyclogermacrene
13	Acorenone
14	Germacrene B
15	p-Cymene
16	Thymol
17	Terpinolene
18	Phytol
19	Camphene
20	Beta-caryophyllene
21	Aromadendrene
22	Viridiflorene
23	Spathulenol
24	(+)-delta-cadinene
25	Hesperidin
26	Umbelliferone
27	1-Ethylpiperidine
Control	Regorafenib

Druglikeness and pharmacokinetics

The Lipinski rule must be followed to choose lead candidates with enhanced potential for oral drug administration. Among the analyzed compounds, those with a single violation of Lipinski's criteria were considered acceptable for further investigation. Twelve compounds possess one violation. In total, 25 compounds met Lipinski's rule criteria for druglikeness. The druglikeness of these phytochemicals is presented in Table 2. Three of Lipinski's laws were violated by diosmin and hesperidin due to their greater number of hydrogen bond acceptors and donors. These violations were indicative of potential challenges in achieving optimal oral bioavailability for these compounds. As a result, they were not subjected to additional investigation. This selection process ensures that the compounds possess favorable physicochemical properties, increasing their potential as lead candidates for drug development.

**Table 2:** Druglikeness of phytochemicals in Conium maculatum MW: molecular weight; NHBD: number of hydrogen bond donor; NHBA: number of hydrogen bond acceptor; RB: rotatable bond; MR: molar refractivity; TPSA: topological polar surface area

S. no.	Phytochemicals	MW	NHBD	NHBA	RB	LogP	MR	TPSA	Lipinski's rule of five violations	Bioavailability score
1	Arabinose	150.13	4	5	0	-2.58	29.77	90.15	0	0.55
2	Caffeic acid	180.16	3	4	2	1.09	47.16	77.76	0	0.56
3	Chlorogenic acid	354.31	6	9	5	-0.75	83.5	164.75	1	0.11
4	Conhydrine	143.23	2	2	2	0.52	46.33	32.26	0	0.55
5	Coniine	127.23	1	1	2	1.55	45.17	12.03	0	0.55
6	Diosmin	608.54	8	15	7	-1.09	143.82	238.2	3	0.17
7	Falcarinol-one	244.37	1	1	8	4.01	80.37	20.23	1	0.55
8	Falcarinone	242.36	0	1	8	4.21	79.41	17.07	1	0.55
9	Gamma-coniceine	125.21	0	1	2	2.03	45.47	12.36	0	0.55
10	Pseudoconhydrine	143.23	2	2	2	0.52	46.33	32.26	0	0.55
11	Myrcene	136.23	0	0	4	3.48	48.76	0	0	0.55
12	Bicyclogermacrene	204.35	0	0	0	4.73	68.78	0	1	0.55
13	Acorenone	220.35	0	1	1	3.98	69.46	17.07	0	0.55
14	Germacrene B	204.35	0	0	0	5.18	70.68	0	1	0.55
15	p-Cymene	134.22	0	0	1	3.12	45.99	0	1	0.55
16	Thymol	150.22	1	1	1	2.82	48.01	20.23	0	0.55
17	Terpinolene	136.23	0	0	0	3.45	47.12	0	0	0.55
18	Phytol	296.53	1	1	13	6.36	98.94	20.23	1	0.55
19	Camphene	136.23	0	0	0	3	45.22	0	1	0.55
20	Beta-caryophyllene	204.35	0	0	0	4.73	68.78	0	1	0.55
21	Aromadendrene	204.35	0	0	0	4.27	67.14	0	1	0.55
22	Viridiflorene	204.35	0	0	0	4.42	67.14	0	1	0.55
23	Spathulenol	220.35	1	1	0	3.39	68.34	20.23	0	0.55
24	Delta-cadinene	204.35	0	0	1	4.73	69.04	0	1	0.55
25	Hesperidin	610.56	8	15	7	-1.48	141.41	234.29	3	0.17
26	Umbelliferone	162.14	1	3	0	1.5	44.51	50.44	0	0.55
27	1-ethylpiperidine	113.2	0	1	1	1.11	40.46	3.24	0	0.55
Control	Regorafenib	482.82	3	8	9	6.88	112.44	92.35	0	0.55

The GIA was found to be higher for caffeic acid, conhydrine, coniine, falcarinol-one, falcarinone, gamma-coniceine, pseudoconhydrine, acorenone, thymol, spathulenol, and umbelliferone. Moreover, conhydrine, coniine, falcarinol-one, falcarinone, gamma-coniceine, pseudoconhydrine, myrcene, acorenone, p-cymene, thymol, terpinolene, camphene, aromadendrene, spathulenol, and umbelliferone were identified as being capable of permeating the BBB. Diosmin, phytol, and hesperidin were found to be substrates of p-glycoprotein (P-gp), a protein responsible for drug efflux. However, none of the remaining 24 compounds were identified as substrates of P-gp. The potential interactions with cytochrome P450 isoenzymes, including CYP1A2, CYP2C19, CYP2C9, CYP2D6, and CYP3A4, were also evaluated. It was observed that falcarinol-one, falcarinone, thymol, aromadendrene, and umbelliferone exhibited inhibition of the CYP1A2 isoenzyme. Bicyclogermacrene, acorenone, beta-caryophyllene, aromadendrene, viridiflorene, spathulenol, and delta-cadinene were identified as inhibitors of the CYP2C19 isoenzyme. Similarly, falcarinol-one, bicyclogermacrene, acorenone, germacrene B, terpinolene, phytol, camphene, beta-caryophyllene, aromadendrene, viridiflorene, and delta-cadinene were found to be inhibitors of the CYP2C9 isoenzyme. In addition, p-cymene was identified as an inhibitor of the CYP2D6 isoenzyme. None of the compounds displayed inhibitory activity against the CYP3A4 isoenzyme. Table 3 provides an overview of the pharmacokinetic properties of the phytochemicals, highlighting their absorption, BBB permeability, p-glycoprotein substrate status, and interactions with various cytochrome P450 isoenzymes.

**Table 3:** Pharmacokinetic properties of the phytochemicals p-gp: P-glycoprotein; GIA: absorption in the gastrointestinal tract; BBB: blood-brain barrier

S. no.	Phytochemicals	GIA	BBB permeant	p-gp substrate	CYP1A2 inhibitor	CYP2C19 Inhibitor	CYP2C9 inhibitor	CYP2D6 inhibitor	CYP3A4 inhibitor
1	Arabinose	Low	No	No	No	No	No	No	No
2	Caffeic acid	High	No	No	No	No	No	No	No
3	Chlorogenic acid	Low	No	No	No	No	No	No	No
4	Conhydrine	High	Yes	No	No	No	No	No	No
5	Coniine	High	Yes	No	No	No	No	No	No
6	Diosmin	Low	No	Yes	No	No	No	No	No
7	Falcarinol-one	High	Yes	No	Yes	No	Yes	No	No
8	Falcarinone	High	Yes	No	Yes	No	No	No	No
9	Gamma - coniceine	High	Yes	No	No	No	No	No	No
10	Pseudoconhydrine	High	Yes	No	No	No	No	No	No
11	Myrcene	Low	Yes	No	No	No	No	No	No
12	Bicyclogermacrene	Low	No	No	No	Yes	Yes	No	No
13	Acorenone	High	Yes	No	No	Yes	Yes	No	No
14	Germacrene B	Low	No	No	No	No	Yes	No	No
15	p-cymene	Low	Yes	No	No	No	No	Yes	No
16	Thymol	High	Yes	No	Yes	No	No	No	No
17	Terpinolene	Low	Yes	No	No	No	Yes	No	No
18	Phytol	Low	No	Yes	No	No	Yes	No	No
19	Camphene	Low	Yes	No	No	No	Yes	No	No
20	Beta-caryophyllene	Low	No	No	No	Yes	Yes	No	No
21	Aromadendrene	Low	Yes	No	Yes	Yes	Yes	No	No
22	Viridiflorene	Low	No	No	No	Yes	Yes	No	No
23	Spathulenol	High	Yes	No	No	Yes	No	No	No
24	Delta-cadinene	Low	No	No	No	Yes	Yes	No	No
25	Hesperidin	Low	No	Yes	No	No	No	No	No
26	Umbelliferone	High	Yes	No	Yes	No	No	No	No
27	1-ethylpiperidine	Low	No	No	No	No	No	No	No
Control	Regorafenib	Low	No	No	Yes	Yes	Yes	Yes	Yes

Analysis of molecular docking

**Figure 2:**
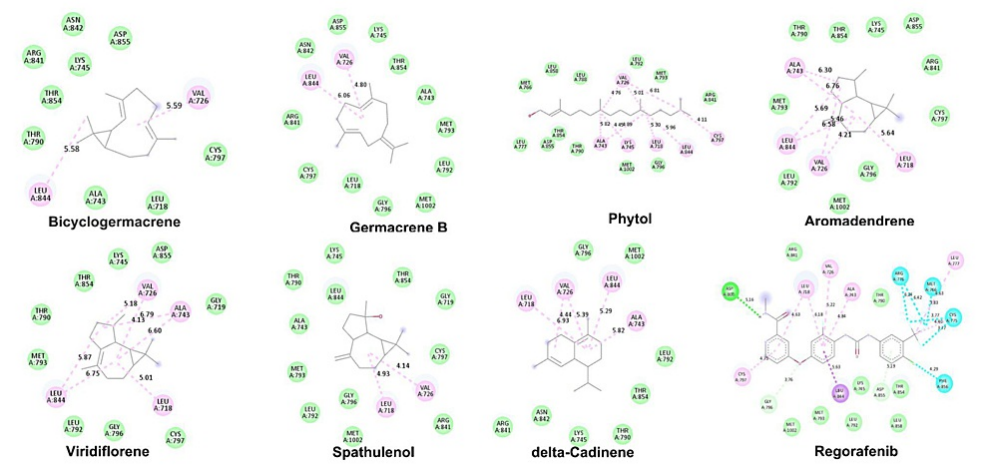
2D docked structure of the ligands interacting with EGFR EGFR: epidermal growth factor receptor

Table 4 provides detailed information on the binding affinity and amino acid interactions between EGFR and the bioactive compounds. The binding affinities of phytol, caffeic acid, falcarinone, bicyclogermacrene, delta-cadinene, acorenone, beta-caryophyllene, spathulenol, umbelliferone, germacrene B, aromadendrene, and viridiflorene ranged from -6.5 to 8 Kcal/mol, indicating their potential as ligands for targeting EGFR. The amino acid interactions observed between these compounds and EGFR contribute to their binding and potential activity against the target. Figure 2 visually represents the 2D docked structure of the selected ligands interacting with EGFR with the best binding affinity, providing insights into their binding orientations and potential interactions with key residues in the binding site. This structural information enhances our understanding of the ligand-receptor interactions and can aid in further optimization and drug design. Based on their favorable binding affinities, these 12 compounds were further analyzed for their potential toxicological effects and safety profiles.

**Table 4:** Binding affinity and amino acid interactions between EGFR and the bioactive compounds EGFR: epidermal growth factor receptor

S. no.	Phytochemicals	Binding affinity (Kcal/mol)	No. of H bond	Hydrogen bond	Carbon hydrogen / Pi-anion / Pi-sulfur / Pi-Pi stacked / Pi-alkyl bond	Van der waals
1	Arabinose	-5.8	3	Phe856(3.26), Thr845(4.92), Asp855(3.99)	Thr790	Val769, Arg776, Cy775, Leu777, Leu788, Met766, Leu858
2	Caffeic acid	-6.7		Lys7445(4.27), Asp855(3.83), Cys775(4.52) Met766(4.26), Phe856(3.38)	Leu777	Val726, Thr854, Leu858, Val769, Arg776, Thr790
3	Chlorogenic acid	-8.4	2	Met793(3.6), Thr790(4.52)	Leu844, Ala743, Val726, Leu718	Gly796, Cys797, Met1002, Leu792, Thr854, Leu777, Cys775, Arg776, Leu788, Met766, Leu858, Asp855, Lys745
4	Conhydrine	-5.3	1	Thr854(4.22)	Leu858, Leu788, Lys745 ,Phe856, Leu777, Met766	Arg776, Cys775, Thr790, Asp855
5	Coniine	-5.2	-	-	Met766, Phe856, Leu777, Leu788, Lys745	Arg776, Cys776, Thr790, Val726, Asp855, Thr854
6	Falcarinol-one	-6.2	2	Leu788(5.20), Ala743(3.72)	Leu777, Val726, Leu718, Leu792, Leu844	Ile744, Ile789, Thr790 ,Thr854, Lys745, Met1002, Cys797, Gly796, Met793
7	Falcarinone	-6.9	1	Met793(4.30)	Phe856, Met766, Lys745, Leu777, Leu788, AlA743, Val726	Arg776, Asp855, Cys775, Thr790, Leu792, Leu844, Gly796, Leu718, Met1002, Leu858, Thr854
8	Gamma- Coniceine	-5.2	-	-	Lys745, Leu788, Met766, Phe856	Thr790, Thr854, Cys775, Arg776, Asp855, Leu777, Leu858
9	Pseudoconhydrine	-5.6	-	-	Lys745	Leu777, Cys775, Arf776, Val769, Met766, Phe856, Asp855. Thr854, Thr790, Leu858, Leu788
10	Myrcene	-5.4	-	-	Val726, Lys745, Ala743, Leu777, Met766, Cys775, Phe856	Thr790, Asp855, Thr854, Leu788, Arg776
11	Bicyclogermacrene	-6.9	-	-	Val726, Leu844, Leu718, Ala743	Ala743, Leu718, Cys797, Thr790, Thr854, Lys745, Arg841, Asn842, Asp855
12	Acorenone	-7.4	-	-	Thr790, Val726, Ala743, Lys745	Leu788, Leu858, Leu777, Met766, Asp855, Thr854, Cys775, Thr790, Leu844
13	Germacrene B	-7.5	-	-	Leu844, Val726	Arg841, Cys797, Leu718, Gly796, Met1002, Leu792, Met793, Ala743, Thr854 ,Lys745, Asp855, Asn842
14	p-Cymene	-5.8	-	-	Met766, Leu777	Leu858, Asp855, Thr854, Lys745, Val726, Ile789, Thr790, Ala743, Leu788
15	Thymol	-6	1	Asp855(2.80)	Leu777, Met766, Phe856	Thr790, Cys775, Arg776, Thr854, Leu858, Lys745
16	Terpinolene	-6	-	-	Val726, Leu844, Leu718, Ala743	Gly796, Met1002, Met793, Leu792, Thr854, Lys745, Asp855
17	Phytol	-6.6	-	-	Val725, Ala743, Lys 745. Leu844, Cys797	Met766. Leu858, Leu788, Leu792, Met793, Arg841, Gly796, Met1002, Thr790, Thr854, Asp855, Leu777
18	Camphene	-5.6	-	-	Leu788, Lys745	Thr845, Asp855, Leu777, Leu858, Thr790. Ala743, Val726, Leu844
19	beta-Caryophyllene	-7.4	-	-	Leu844, Val726	Leu718, Ala743, Thr790, Lys745, Asp855, Thr854, Arg841, Asn842, Cys797
20	Aromadendrene	-7.7	-	-	Ala743, Leu844, Val726, Leu718	Thr790, Thr854, Lys745, Asp855, Arg841, Cys797, Gly796, Met1002, Leu792. Met793
21	Viridiflorene	-7.9	-	-	Leu844, Leu718, Ala743, Val726	Met793, Thr790, Thr854, Lys745, Asp855, Gly719, Cys797, Gly796, Leu792
22	Spathulenol	-7.4	-	-	Leu718, Val726	Arg841, Cys797, Gly719, Thr854, Lys745, Leu844, Thr790, Ala743, Met793, Leu792, Gly796, Met1002
23	delta-Cadinene	-7.2			Leu718, Val726, Leu844, Ala743	Gly796, Met1002, Leu792, Thr854, Thr790, Lys745, Asn842, Arg841
24	Umbelliferone	-7.4	1	Met766(3.22)	Leu788, Lys745, Leu777, Phe856	Thr790, Cys775, Arg776, Val769, Thr854, Asp855, Leu858
25	1-Ethylpiperidine	-4.7	1	Thr854(4.82)	Leu777, Met766, Phe856	Asp855, Leu858, Lys745, Leu788, Thr790, Arg776, Cys775
Control	Regorafenib	-9.7	1	Asp800(5.16)	Lys797, Leu718, Val726, Ala743, Leu844, Phe856, Cys775, Met766, Arg776, Leu777	Arg841, Gly796, Met1002, Thr790, Met793, Leu792, Lys745, Asp855, Leu858, Thr854

Toxicity studies

Table 5 provides the toxicity profiles of the compounds investigated in this study. Among the compounds analyzed, bicyclogermacrene, germacrene B, phytol, aromadendrene, viridiflorene, spathulenol, and delta-cadinene demonstrated no predicted toxicity in organs and other toxicological endpoints. These compounds exhibited a favorable toxicological profile, suggesting their potential as safer options for further analysis. The acute toxicity class of these compounds was determined to be 5, indicating low acute toxicity. The lethal dose (LD50) ranges for these compounds were estimated to be between 3900 and 5300 mg/kg. These values provide insights into the dosage range. The remaining compounds in the study showed positive results for various toxicological endpoints, indicating potential toxic effects. Considering these findings, the compounds exhibiting toxicity were excluded from further analysis. 

**Table 5:** Toxicity profiles of the phytochemicals LD: lethal dose; Act. Tox. Cla.: acute toxicity class

No	Phytochemicals	LD 50 (mg/kg)	Act. Tox. Cla.	Hepatoxicity	Carcinogenicity	Immunotoxicity	Mutagenicity	Cytotoxicity
1	Caffeic acid	2989	5	Inactive	Active	Inactive	Inactive	Inactive
2	Chlorogenic acid	5000	5	Inactive	Inactive	Active	Inactive	Inactive
3	Falcarinone	5000	5	Inactive	Inactive	Active	Inactive	Inactive
4	Bicyclogermacrene	5300	5	Inactive	Inactive	Inactive	Inactive	Inactive
5	Acorenone	2450	5	Inactive	Inactive	Active	Inactive	Inactive
6	Germacrene B	4390	5	Inactive	Inactive	Inactive	Inactive	Inactive
7	Phytol	5000	5	Inactive	Inactive	Inactive	Inactive	Inactive
8	Beta-caryophyllene	5300	5	Inactive	Inactive	Active	Inactive	Inactive
9	Aromadendrene	5000	5	Inactive	Inactive	Inactive	Inactive	Inactive
10	Viridiflorene	5000	5	Inactive	Inactive	Inactive	Inactive	Inactive
11	Spathulenol	3900-	5	Inactive	Inactive	Inactive	Inactive	Inactive
12	Delta-cadinene	4390	5	Inactive	Inactive	Inactive	Inactive	Inactive
13	Umbelliferone	10000	6	Inactive	Active	Inactive	Inactive	Inactive
Control	Regorafenib	800	4	Active	Inactive	Active	Inactive	Active

Analysis of dynamic simulations

**Figure 3:**
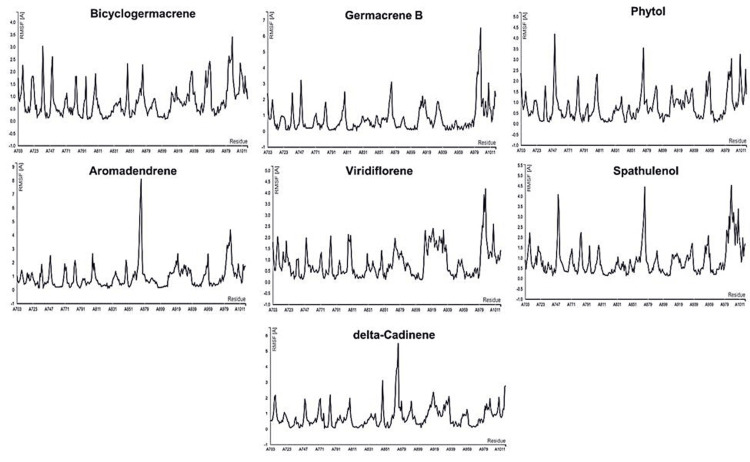
Root mean square fluctuation (RMSF) of hits from CABS-flex

Bicyclogermacrene, germacrene B, phytol, aromadendrene, viridiflorene, spathulenol, and delta-cadinene were the identified hits in this study. The dynamic simulation was carried out for these seven compounds. Figure 3 illustrates the fluctuation plots of the ligand-target complex. From the plots, minimal fluctuations in the residues indicate a high degree of stability. The RMSF values for all complexes were consistently below 10 Å, suggesting that the protein-ligand interactions remained stable under physiological conditions. This finding indicates that the complexes maintained their structural integrity throughout the dynamic simulation. These results further support the notion that the identified protein-ligand complexes have the potential for stable and reliable interactions, making them promising candidates for further investigation in drug discovery and development.

Bioavailability radar and molecular targets

**Figure 4:**
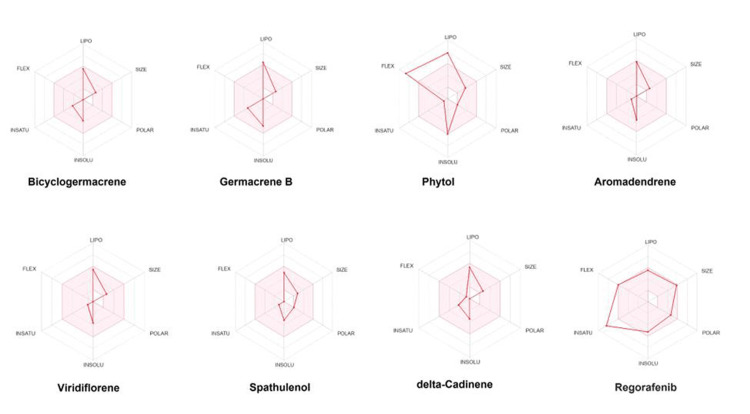
Radar plots for the identified leads

**Figure 5:**
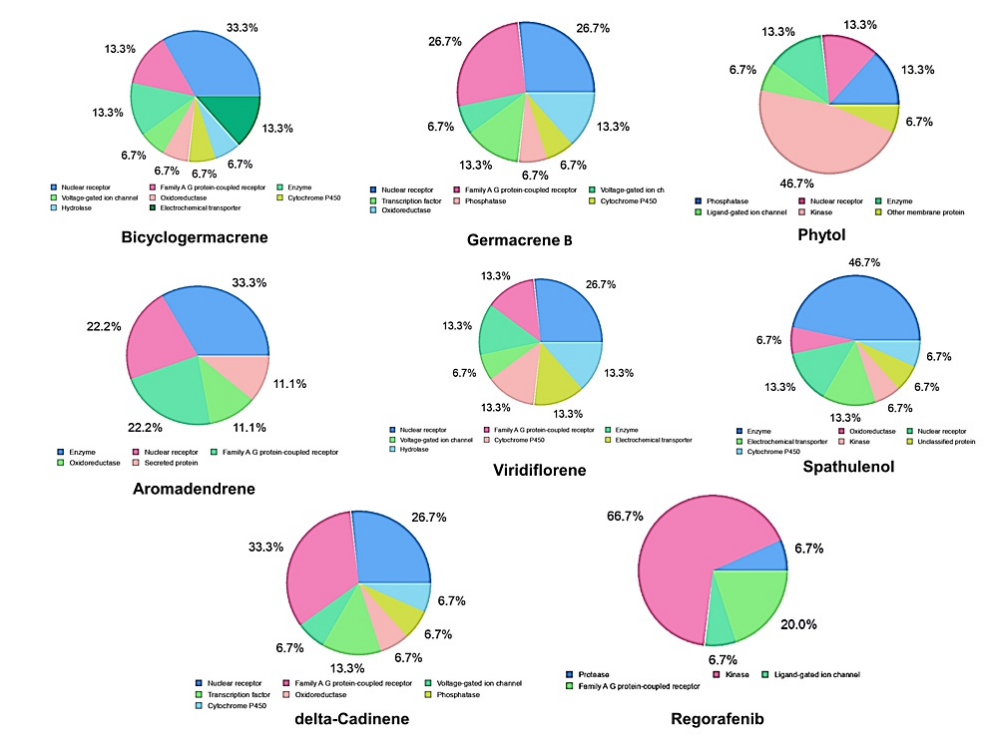
Molecular targets of the identified hits

Figure 4 depicts the radar plots for bicyclogermacrene, germacrene B, phytol, aromadendrene, viridiflorene, spathulenol, and delta-cadinene. These plots highlight the optimal range for each compound, represented by the pink color. Compounds falling within the pink region of the radar plot indicate favorable oral bioavailability. Notably, all compounds, except phytol, demonstrated favorable oral bioavailability based on their positioning within the pink region. Radar plots are useful visualization tools that provide a comprehensive overview of multiple parameters or characteristics of a compound. Figure 5 illustrates the molecular targets of the identified hits. Understanding the molecular targets is crucial for elucidating the potential therapeutic applications of the compounds. The oral bioavailability of the lead compounds was assessed using a bioavailability radar plot, which was previously reported by Kadri and Aouadi [25]. This analysis indicated that the identified compounds from *C. maculatum* were orally bioavailable, further supporting their potential as therapeutic agents. In addition, molecular target screening of the phytochemicals was performed to identify their potential targets.

## Discussion

The present study utilized a comprehensive in silico approach to explore the potential of phytochemicals derived from *C. maculatum* as inhibitors of the EGFR in the context of mCRC. A total of 25 phytochemicals from *C. maculatum* met Lipinski's rule criteria, indicating their potential as drug-like properties. Among these, 12 compounds demonstrated favorable binding affinities to the target protein. Furthermore, seven of these compounds exhibited no predicted toxicological concerns. In addition, the bioavailability radar indicated that these lead compounds could be administered orally. This virtual screening process yielded seven lead compounds from a pool of 27 phytochemicals. 

The selection of *C. maculatum* for investigation was based on its rich phytochemical content and the potential anticancer activity associated with its compounds. For the in silico druglikeness screening and pharmacokinetics, the analysis was conducted to evaluate the suitability of the phytochemicals for further investigation. Previous studies have employed similar screening approaches in other plant species, such as *Ipomoea mauritiana,* to assess the druglikeness and pharmacokinetic properties of their phytochemicals [26]. The results of these studies provided valuable references for the current research. Twenty-five compounds from *C. maculatum* were identified with favorable druglikeness and pharmacokinetic properties, and further molecular docking studies were performed to evaluate the binding affinity of these compounds toward EGFR. A similar interaction between EGFR and flavonoids has been previously investigated by Sepay et al. [27]. The authors employed structure-based approaches to design new EGFR inhibitors in their study. These findings highlight the significance of molecular docking studies in predicting the potential of compounds as inhibitors of specific molecular targets [27]. 

Molecular dynamics simulations performed on these seven protein-ligand complexes in our study revealed stable and reliable interactions, underscoring their promise as potential candidates. Nag et al. [28] studied the stability of human papillomavirus (HPV)-induced cervical cancer proteins using a CABS-flex 2.0 server. The researcher suggested that the interactions between the protein-ligand complex were strong and robust, allowing for a stable binding throughout the simulation [28].

In another study conducted by Tumski and Tumskaia [29], the researchers utilized the CABS-flex 2.0 server to investigate the stability of the SARS CoV-2 main protease complexed with ligands. The researchers concluded that the protein-ligand interactions influenced the overall stability of the complexes. Sundar et al. [30] utilized a CABS-flex 2.0 web server for studying the repurposing of existing antiviral drugs targeting dengue proteins. The result of the study concluded that repurposed drug exhibited minimal fluctuations in the residues, suggesting that the protein-ligand complexes were stable [30]. Furthermore, a molecular target screening of the phytochemicals was conducted to identify potential interaction targets. The pie chart generated in this research displays the distribution of the potential molecular targets of the leads. Our study is consistent with a prior investigation, suggesting that our findings align with those from the earlier research. Since this analysis is based only on computational approach, further experimental validation is necessary.

Limitations and future recommendations

While this study provides a promising starting point for the development of potential anticancer compounds from natural sources, it is essential to recognize the limitations associated with its computational nature and preliminary findings. Our entire study relies on computational techniques, which may not fully represent the complex biological interactions. The results are based on predictions and simulations and require rigorous experimental validation to confirm the compound's effectiveness and safety in treating mCRC. Furthermore, the identified lead may require further optimization processes to enhance efficacy and safety. Our study also highlighted potential lead compounds, but experimental validation, such as cell-based assays or animal studies, is required to confirm their efficacy and safety in a real biological context.

## Conclusions

The virtual screening of phytochemicals derived from *C. maculatum* as potential inhibitors of EGFR in mCRC has provided valuable insights into the potential of natural compounds as anticancer agents. From 27 compounds screened, seven highly potential leads have been identified. These lead compounds showed comparable activity to the control drug regorafenib, a known EGFR inhibitor. This suggests their potential as alternative therapeutic agents for mCRC, targeting the EGFR pathway. In addition, mechanistic studies should be conducted to elucidate the specific molecular mechanisms underlying the inhibitory effects of the leads.
